# A Reliable Nonhuman Primate Model of Ischemic Stroke with Reproducible Infarct Size and Long-term Sensorimotor Deficits

**DOI:** 10.14336/AD.2022.0722

**Published:** 2023-02-01

**Authors:** Xiao Lin, Hua Wang, Shengwei Huang, Lefu Chen, Su Yang, Peiqi Zhao, Zhongxiao Lin, Jianjing Yang, Linhui Ruan, Haoqi Ni, Kankai Wang, Min Wen, Kunlin Jin, Qichuan Zhuge

**Affiliations:** ^1^Department of Neurosurgery, First Affiliated Hospital of Wenzhou Medical University, Wenzhou, China.; ^2^Zhejiang Provincial Key Laboratory of Aging and Neurological Disorder Research, Wenzhou Medical University, Wenzhou, China.; ^3^Department of Neurology, Guangzhou First People's Hospital, Guangzhou, China.; ^4^Department of Pharmacology and Neuroscience, University of North Texas Health Science Center, Fort Worth, TX 76107, USA

**Keywords:** ferric chloride, cynomolgus macaque, nonhuman primate, ischemic stroke, middle cerebral artery

## Abstract

A nonhuman primate model of ischemic stroke is considered as an ideal preclinical model to replicate various aspects of human stroke because of their similarity to humans in genetics, neuroanatomy, physiology, and immunology. However, it remains challenging to produce a reliable and reproducible stroke model in nonhuman primates due to high mortality and variable outcomes. Here, we developed a focal cerebral ischemic model induced by topical application of 50% ferric chloride (FeCl_3_) onto the MCA-M1 segment through a cranial window in the cynomolgus monkeys. We found that FeCl_3_ rapidly produced a stable intraarterial thrombus that caused complete occlusion of the MCA, leading to the quick decrease of the regional cerebral blood flow in 10 min. A typical cortical infarct was detected 24 hours by magnetic resonance imaging (MRI) and was stable at least for 1 month after surgery. The sensorimotor deficit assessed by nonhuman primate stroke scale was observed at 1 day and up to 3 months after ischemic stroke. No spontaneous revascularization or autolysis of thrombus was observed, and vital signs were not affected. All operated cynomolgus monkeys survived. Our data suggested that FeCl_3_-induced stroke in nonhuman primates was a replicable and reliable model that is necessary for the correct prediction of the relevance of experimental therapeutic approaches in human beings.

Stroke remains the second leading cause of death and leading cause of serious long-term disability worldwide [1, 2]. Ischemic stroke accounts for more than 70% of all strokes [3, 4]. The intravenous use of tissue plasminogen activator (tPA) permitted by the Food and Drug Administration (FDA) to dissolve thrombus is the only drug for acute ischemic stroke, but its use is limited by several factors including short therapeutic window, selective efficacy, and subsequent hemorrhagic complications [5, 6]. Therefore, only fewer than 15% stroke patients can benefit from tPA, and there is an urgent need to develop more effective, safe, and feasible treatment methods for ischemic stroke [7].

For the last two decades, although evidence has shown that neuroprotective drugs are effective for treating acute stroke in animal models, none of the neuroprotective agents has been proven to be clinically beneficial [8]. Many possible reasons may cause the failures. Lack of satisfactory animal models resembling human ischemic stroke could be a major reason for the failure to develop successful neuroprotective drugs for ischemic stroke. Nonhuman primates (NHPs) have been suggested as an ideal animal model for preclinical, translational stroke research by the Stroke Therapy Academic Industry Roundtable (STAIR) committee due to the fact that nonhuman primates have more similarity to humans in genetics, neuroanatomy, physiology and immunology than rodents have [9, 10]. A variety of approaches have been used to develop ischemic stroke in NHPs in last 30 years. The middle cerebral artery (MCA) is the most common artery involved in stroke in clinical setting, which can be produced by endovascular approaches and craniotomy. Therefore, early studies used arterial clips or coagulation to occlude blood flow of the MCA through transorbital approach and transcranial approach [11, 12]. The main limitations of the models include that it is difficult to be operated and associated with a high risk of mortality, along with the irreversible damage of the blood vessels [13]. Late, Kito et al., develop an experimental model of thromboembolic stroke by injection of an autologous blood clot to the MCA via the internal carotid artery in cynomolgus monkeys [14], which closely mimics thromboembolic or thrombotic cerebrovascular occlusion in patients. However, the major challenges of these models in NHPs are to control exactly the occlusion site and recanalization of the vessel. With the advancement of digital subtraction angiography (DSA) technology, transient MCA occlusion in NHPs is generally induced with a retrievable tool, such as a microcatheter, an inflatable balloon, and a microcoil, under DSA [15, 16]. The completed reperfusion is achieved when the retrievable tool is withdrawn in NHPs, developing infarct size and neurological impairment. However, blood vessel rupture and bleeding are the major concerns. In addition, these methods are limited for thrombolysis treatment. Notably, Freret et al invented the NHP stroke model using intraluminal thread to block the MCA through internal carotid artery in 2007 [17]. The method is relatively easy to be operated, along with low mortality, which however cannot be used in thrombolysis studies and may cause hemorrhage. Several other methods, such as photothrombosis and endothelin-1 injection, have been used for producing NHP stroke models [18]. Still, the resulting thrombi of these methods do not produce a satisfactory infarct size and their reproducibility is poor [19-21]. Therefore, there is an urgent need to produce a reliable and reproducible stroke model in NHPs, with stable outcomes, low mortality and physiological and pathological changes consistent with human ischemic stroke.

Ferric chloride (FeCl_3_) has been studied to produce ischemic stroke model in rodents without obvious toxic effects [22-24]. The thrombus induced by FeCl_3_ is similar to human thrombus, which can thus be dissolved by thrombolytic drugs. we sought to adapt this approach to nonhuman primates. In this study, we established a NHP stroke model induced by topical application of FeCl_3_ onto the M1 segment of the MCA. The successful embolization of the blood vessel and a typical cortical infarct were confirmed by magnetic resonance angiography (MRA) and magnetic resonance imaging (MRI). The stable infarction and sensorimotor deficits were observed in 3 months after surgery, and all NHPs survived. Our data suggest that FeCl_3_-induced MCAO in NHPs is feasible and mimic the pathogenesis of ischemic stroke patients. Therefore, it is ideal for studying the effect of promising therapies that require proper thrombolysis treatment.

## MATERIALS AND METHODS

### Animals

**Figure 1.**
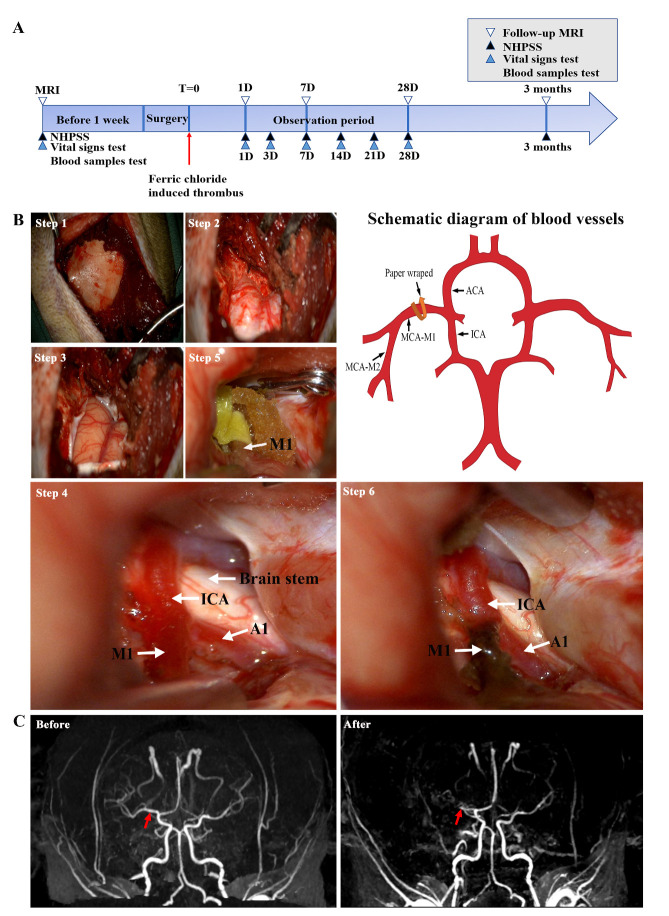
Schematic diagram of the timeline of the ischemic stroke and the MCAO surgical procedure. (A) Experimental design. MRI, NHPSS, vital signs test and blood samples test were performed as indicated. (B) Procedure of cynomolgus monkey exposure of MCA and its branches under the microscope (left panel) and illustration of blood vessels (right panel). Step 1: Excise the skin and subcutaneous tissue to expose the skull. Step 2: Expose the dura mater after removing the bone window. Step 3: After cutting open the meninges, reveal the brain tissue and lateral fissure vessels. Step 4: Expose the deep blood vessels after separating the brain tissue. Step 5: Temporarily clip upstream of the MCA and wrap filter paper around the MCA M1 segment for 10 minutes. Step 6: Remove the filter paper to reveal degeneration of blood vessels and intravascular thrombosis. The white arrow indicates the artery. (C) MRA in a macaque before and 24 hours after surgery. The red arrow indicates the embolism. ICA: internal carotid artery. MCA: Middle Cerebral Artery. MCA-M1: M1 segment of the middle cerebral artery. MCA-M2: M2 segment of the middle cerebral artery. ACA: Anterior cerebral artery. A1: A1 segment of the anterior cerebral artery.

Cynomolgus monkeys (10-11-years-old, male), weighing about 10.0 ± 0.5 kg, were purchased from the Wincon Theracells Biotechnologies (Nanning, China). Each animal was individually kept in a cage, under 12-hour light/dark cycle with room temperature between 18 and 26°C, and humidity between 40 and 70%. Animals were fed twice per day and supplemented with fresh fruits and vegetables once daily. All surgical and experimental procedures were reviewed and approved by the Institutional Animal Care and Use Committee (permit number W00186) of the Institute of Medical Laboratory Animals, Chinese Academy of Medical Sciences, and were implemented in accordance with the "Guidelines for the Protection and Use of Laboratory Animals". Before FeCl_3_-induced MCAO one week, each monkey underwent MRA to confirm intracranial vascular development. As a result, one of the cynomolgus monkeys was excluded due to the absence of one middle cerebral artery. The intracranial vascular structures of the remaining three experimental animals were intact and could continue to FeCl_3_-induced MCAO. Animals were compared with their own preoperative results. Preoperative and postoperative examinations were performed by MRI, nonhuman primate post-stroke score test, vital sign measurement, and blood tests (Fig. 1A).

### FeCl_3_-induced dMCAO

Thrombus was induced by a 50% FeCl_3_-soaked filter paper strip on the tunica adventitia vasorum over the M1 segment of the distal MCA for 10-minutes. After successful anesthesia with ketamine 0.1 mg/kg and tracheal intubation, the head of the monkey was placed in a lateral position, and anesthesia was maintained with 2% isoflurane vaporized in 100% oxygen. A cotton pad was placed under the head and the head was fixed in a clamp. An arterial line was established for blood pressure monitoring throughout surgery to maintain a mean arterial blood pressure of 60-80 mmHg. The operation was conducted by neurosurgeons.

An oblique incision was made on the selected side from the midpoint to the top of the zygomatic arch. A high frequency electric knife (10 W) was used to incise the subcutaneous, superficial temporal fascia and the fascia attached to the surface of the zygomatic arch. The temporalis muscles together with part of the temporal fossa muscles were dissected to clearly expose the blood vessels (Fig. 1B, Step 1). Hemostasis was secured using bipolar electrocoagulation (16 W). A burr hole of about 2 cm was made with a drill around the keyhole on the surface of the skull, and the bone was removed with a sharp-nose rongeur to a diameter of 3.0-3.5 cm. The surgical area was further enlarged by removal of the bone at the medial side of the sphenoid ridge near the anterior clinoid process. After reaching the dura reflection of the sphenoid ridge, a drill was used to smoothen the sphenoid ridge until it was level with the anterior skull base, and the lateral fissure fully exposed (Fig. 1B, Step 2). Bleeding from the bone was stopped with bone wax, and tissue hemostasis was stopped by bipolar coagulation. The dura mater was picked with a dura hook and excised in a semicircular manner using a size 11 blade and durotomy scissors, with the base at the zygomatic arch. The lateral fissure cistern was opened, and the arachnoid was excised with microscissors to slowly release cerebrospinal fluid, and visualize the lateral fissure vessels (Fig. 1B, Step 3). The internal carotid artery and its branches about 4 mm of the MCA from the origin of the M1 segment to the origin of the M2 segment, were exposed (Fig. 1B, Step 4). After exposing the vessel of interest, soaked saline gauze or patties were used to protect the surrounding brain tissue and blood vessels. Filter paper (2 × 4 mm) was soaked in 50% FeCl_3_ (236489, Sigma, USA, m/v, dissolved in deionized water) for 1 min. By temporarily clipping the upstream blood flow of the MCA, the FeCl_3_-impregnated filter paper was wrapped around the M1 segment of the MCA for about 10 min (Fig. 1B, Step 5). The filter paper and aneurysm clip were then removed. Thrombosis observed under a Zeiss operating microscope (Fig. 1B, Step 6). After the procedure was completed, a gelatin sponge was used to the drape brain surface. The wound was closed, and NHPs were resuscitated and monitored in special cages for follow-up inspections and experiments. Treatment procedures such as immobilization, sedation and anesthesia were performed by veterinary technicians according to standard guidelines.

### MRI

Each experimental monkey was intubated and anesthetized (isoflurane 1.0-1.5% vaporized in 100% oxygen) and MRI scan (a Siemens 3.0T MRI machine with a 32-channel head coil) was performed 1 week before, and 1 day, 7 days, 28 days, and 3 months after surgery. We performed T1-weighted imaging, T2-weighted imaging, fluid attenuated inversion recovery (FLAIR) and MRA of whole brain at slice thickness of 1.5 mm. All images were acquired in DICOM format, and the postoperative cerebral infarction area was qualitatively evaluated.

### Quantification of ischemic damage

The infarct volume was calculated by analyzing the area of the infarct layer by layer, according to the MRI FLAIR results (Sante DICOM Viewer and ImageJ), and the following formula was used for calculated: infarct area of each layer × thickness (1.5 mm) = infarct volume (mm^3^).

### Measurement of vital signs and analysis of blood samples

Vital signs including weight, respiratory rate, heart rate and body temperature were also measured daily. The blood samples were collected from the calf vein 1 week before surgery, and 1, 3, 7, 14, 21 and 28 days after surgery, and then analyzed by blood routine analyzer (BC5390, Mindray, China) for white blood cell count, hemoglobin, platelet count, Na^+^, K^+^, CI^-^ and glucose. The alanine transaminase (ALT), aspartate transaminase (AST), albumin, creatinine was analyzed by blood biochemical analyzer (BS2000, Mindray) for liver function and prothrombin time (PT), activated partial thromboplastin time (APTT), thrombin time (TT) and fibrinogen were determined by coagulation function analyzer (CS5100, Sysmex, Japan) for blood coagulation function.

### Behavioral tests

Neurological outcome was assessed using the NHP stroke scale (NHPSS, Table 1)[25] 1 week before and 1, 3, 7, 14, 21, 28 and 90 days after surgery. The NHPSS is a comprehensive score, which currently includes the stroke scoring system of the National Institutes of Health, and comprises 11 components: state of consciousness, defense response, grip reflex, limb movement, gait, rotation, slow movement, balance, neglect, vision defect/hemianopia, and facial weakness. The sum total of NHPSS scores is 41 points with 0 being normal behavior, and 41 points corresponding to severe neurological impairment. The assessment for our study was done by two investigators, and the average scores were tabulated and calculated.

**Table 1:** Non-human primate stroke score (NHPSS).

Stroke clinical rating scale	Content — points
State of consciousness (0-2)	Normal — 0 Drowsy or apathetic —1 Unconscious--2
Defense reaction (0-2)	Normal — 0 Diminished —1 None — 2
Grasp reflex (right/left) (0-1*2):	Present — 0 Absent —1
Extremity movement (upper/lower, right/left) (0-4 *4):	Normal—0 Asymmetrical use or strength noted —1 Clear, marked weakness — 2 Minimal movement, profound weakness — 3 No voluntary use and no use in response to stimulation — 4
Gait (0-3)	Normal—0 Limping—1 Severely impaired—2 Does not walk (but may crawl) —3
Circling (0-2)	Normal behavior — 0 Noticeable preference to turn to one side — 1 Constant rotation — 2
Bradykinesia (0-2)	None—0 Mild—1 Severe—2
Balance (0-2)	Normal—0 Mildly impaired—1 Profoundly impaired, unable to stand on two feet — 2
Neglect (right/left) (0- 2*2)	No neglect — 0 Extinction of stimulus to one side when presented with simultaneous stimuli — 1 Complete neglect of all stimuli, visual, auditory, and tactile, presented to the affected side — 2
Visual field cut/hemianopsia (right/left) (0-1*2)	None—0 No response to visual stimuli in the affected field. differentiated from neglect by the absence of blinking reflex (does not differentiate Cortical lesion, but diagnoses optic tract or optic radiation injury as opposed to cortical problem — 1
Facial weakness (right/left) (0-2*2)	No weakness — 0 Mild —1 Profound (if central 7th - constant drooling, hanging angle of mouth —2

### Statistical Analysis

Statistical analysis was performed using SPSS version 22.0 and Prism version 7.0. All time-related results were expressed as means ± standard deviation. The sample size and *P* values are provided with each figure. The data normality was determined using a Shapiro-Wilks test. For normally distributed populations of data points, one-way analysis of variance (ANOVA) was used. Results were only considered to be statistically significant at *P* < 0.05.

## RESULTS

In the beginning, we tested different FeCl_3_ concentrations and application durations on the rat common carotid artery, which size is similar to M1 segment of the distal MCA in NHPs. After establishment of a reproducible occlusion of the rat common carotid artery with FeCl_3_, we investigated whether similar results could be achieved in the MCA-M1 in NHPs. We found that topical application of a 50% FeCl_3_-soaked filter paper strip on the duramater over the M1 segment of the MCA in NHPs for 10 min could cause intraarterial thrombosis and total occlusion detected under a microscope. The data were confirmed by MRA analysis 24 hours after the FeCl_3_ application (Fig. 1C, red arrow indicates the embolism), suggesting that topical application of 50% FeCl_3_ induced a stable intra-arterial thrombus and ischemia in NHPs.

**Figure 2.**
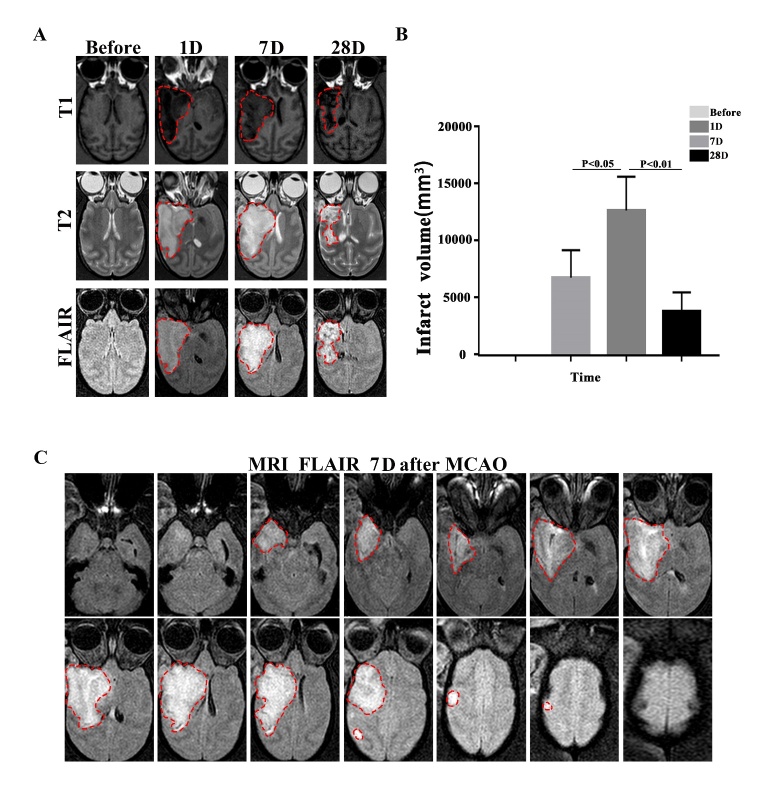
The infarct volume assessed by means of MRI in MCAO cynomolgus monkeys. (A) The brain lesions (highlighted area) were detected by MRI at different time points after M1 MCAO. (B) Quantification of the infarct volume. Cortical infarct volume is calculated based on FLAIR after surgery by image J. One-way ANOVA (**P<0.01, *P<0.05, n=3). (C) A representative image showing FLAIR sequence at 7 days after FeCl_3_-induced MCAO in cynomolgus monkeys. The highlighted area in white is the high signal area under the FLAIR sequence.

Next, we explored whether infarction caused by FeCl_3_-induced MCA-M1 embolization could induce stable and reproducible infarct volume. Infarct volume measured on MRI provides an objective, quantitative measurement of stroke severity, which is less explored in chronic stage of ischemic stroke. Therefore, we used MRI to determine lesion volume 1, 7 and 28 days after surgery (Fig. 2A). We found that all cynomolgus monkeys developed infarcts with similar size. A mean infarct volume was of 6,804±2,415 mm^3^ 1 day after surgery (Fig. 2B) but increased to 12,701±2,966 mm^3^ at 7 days after MCAO. However, the infarct volume decreased to 3,834±1,692 mm^3^ 28 days after stroke. The pattern of evolution of cerebral infarct volume is similar to the clinical findings. To further determine the area involved in the infarct, we analyzed the MRI FLAIR images on postoperative day 7 and found that the temporal lobe, part of the parietal lobe, caudate and putamen of the cerebral hemisphere were the primary sites of brain infarction (Fig. 2C).

**Figure 3.**
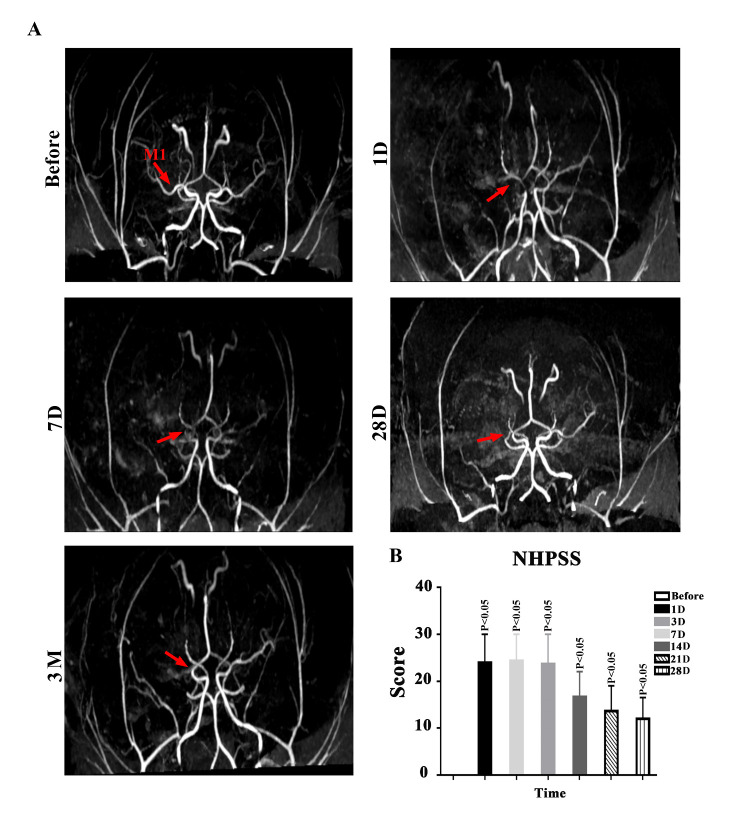
Stable embolization and long-term neurological deficits in NHPs subjected to MCAO. (A) A representative MRA images in cynomolgus monkeys before and 1, 7 and 28 days and 3 months after MCAO. The red arrow indicates the location of induced embolization in the MCA. (B) NHPSS scores during the 3-month observation after FeCl_3_-induced MCA thrombosis in cynomolgus monkeys. One-way ANOVA. (**P<0.01, *P<0.05, n=3).

It is important to know whether autolysis of thrombi or spontaneous revascularization of MCA embolization will occur after FeCl_3_-induced MCAO. To address this, we performed MRA 1, 7, 28 and 90 days after MCAO and found no autolysis of thrombi or spontaneous revascularization after embolization (Fig. 3A).

Then, we asked whether FeCl_3_-induced ischemic stroke could cause sensorimotor deficits, we measured NHPSS scores before and after proximal MCAO (Fig. 3B) and found that sensorimotor deficits were significant on postoperative days 1, 3 and 7, and last at least 3 months after ischemic stroke, although neurological impairment was slightly improved at the chronic stage after stroke in NHPs.

**Table 2:** Vital signs and blood simple tests.

Time after MCA occlusion (day)	Before operation (baseline)	1	3	7	14	21	28
Physiological parameters:							
W (kg)	10.6±1.0	10.4±0.8	10.0±0.8	10.0±0.9	10.0±1.2	9.5±0.9	9.6±0.8
T(°C)	38.4±0.3	36.1±1.3	36.8±0.3	37.7±0.8	36.9±0.8	37.3±0.5	38.2±1.2
HR (beats/min)	123.0±7.2	150.3±56.9	198.7±8.6	178.3±23.5	145.0±49.6	189.7±12.3	157.0±63.9
R (beats/min)	32.7±3.1	29.0±14.9	35.3±5.0	41.0±1.7	33.3±2.9	30.0±7.0	33.0±3.0
Routine blood test:							
WBC (10^9/L)	8.7±3.0	15.2±5.0	14.6±2.3	13.5±4.3	11.1±4.8	8.7±2.2	9.0±2.0
HGB (g/L)	123.3±9.0	135.7±13.2	121.7±7.1	119.7±8.6	107.7±7.4	114.0±11.3	121.7±9.6
PLT (10^9/L)	334.0±55.1	388.3±73.5	402.3±65.0	548.7±158.2	497.3±101.7	563.3±151.9	387.0±100.5
Electrolyte and glucose test:							
K +(mmol/L)	4.0±0.2	3.9±0.7	4.3±0.4	4.6±0.1	4.7±0.9	4.4±0.7	4.5±0.6
Na+(mmol/L)	149.2±1.8	150.2±1.6	154.9±4.8	151.8±3.7	146.5±1.9	147.5±1.7	148.7±1.8
Cl -(mmol/L)	109.3±1.5	106.0±2.9	108.9±6.9	106.4±4.8	105.8±0.4	104.2±1.8	105.4±2.4
Glu(mmol/L)	4.3±0.3	3.3±0.9	3.8±1.0	3.1±0.6	3.2±0.5	3.1±0.2	3.2±0.5
Coagulation function test:							
PT (s)	8.7±0.3	9.2±0.4	8.7±0.4	8.5±0.6	9.3±1.0	8.2±0.3	8.4±0.2
APTT (s)	19.2±1.6	19.6±0.6	16.9±0.6	19.2±2.4	23.4±2.1	21.6±0.4	21.3±0.7
TT (s)	22.1±0.6	21.2±0.8	22.0±1.8	22.0±0.5	23.8±2.5	21.9±0.8	22.0±0.7
FIB(g/L)	2.8±1.0	3.3±0.7	6.0±0.5^**^	4.4±1.5	3.0±0.1	2.5±0.1	2.6±0.3
Liver function test:							
ALT(U/L)	55.5±22.6	149.2±11.5^***^	231.6±17.2^***^	162.3±30.3^***^	54.2±6.3	39.5±1.0	47.8±18.3
AST(U/L)	38.2±13.5	400.7±128.8^***^	408.0±82.5^***^	109.9±18.6	54.8±8.5	48.6±9.7	46.4±14.1
CREA (umol/L)	104.9±19.7	133.0±67.5	114.4±39.2	97.4±18.5	88.8±20.8	84.9±12.5	93.0±12.4
ALB(g/L)	38.2±1.0	38.8±3.1	35.1±2.2	35.8±1.0	35.9±2.7	38.9±1.6	39.1±1.9

Values are the mean ± SD obtained from three monkeys; statistically significant compared to baseline (before operation). One-way ANOVA.

* P<0.05,

** P< 0.01,

*** P< 0.001. Unmarked means no statistical difference. W, weight; T, body temperature; HR, heart rate; R, respiratory rate; WBC, white blood cell; HGB, hemoglobin; PLT: platelets; Glu, glucose; PT, prothrombin time; APTT, activated partial thromboplastin time; TT, thrombin time; FIB, fibrinogen; ALT, alanine transaminase; AST, aspartate transaminase; ALB, albumin; CREA, creatinine.

Finally, we investigated whether FeCl_3_ application affected physiological or biochemical functions in NHPs after ischemic stroke through measuring their vital signs and blood samples (Table 2). All surgical NHPs survived and no significant differences in heart rate, respiratory rate, body weight, and body temperature were observed before and after stroke. Similarly, no significant changes in white blood cells, platelets, hemoglobin, electrolytes, and glucose. Coagulation profile before and after surgery showed no changes in PT, APTT, and TT but slight increase in fibrinogen on postoperative day 3, and normalization on day 7. Liver function abnormalities (AST and ALT levels) were seen on the first and third days after stroke but returned to baseline after 7 days. The data suggest that vital signs were not affected by FeCl_3_ application.

## DISCUSSION

In this study, we found that topical application of 50% FeCl_3_ rapidly produced intraarterial thrombosis and caused completed occlusion of MCA in cynomolgus monkeys. The infarct size is relatively large. No spontaneous revascularization was found at the chronic stage after ischemic stroke, and thus infarct volume is stable and reproducible at least for one month. Significant sensorimotor deficits were observed at acute, subacute and chronic stages after MCAO. Importantly, all surgical cynomolgus monkeys are survival and no vital signs and biochemical functions are affected. Our findings suggested that FeCl_3_ could induce a powerful NHP stroke model with reproducible infarct size and long-term sensorimotor deficits, along with a long survival period.

Ferric chloride was first used to induce MCA thrombosis in mice, which can rapidly reduce a regional cerebral blood flow in 10 min after topical application of FeCl_3_ [26]. In the present study, we modified the FeCl_3_ concentration and the application duration to produce a defined thrombotic occlusion of the MCA-M1 segment in cynomolgus monkeys. The MCA-M1 segment in cynomolgus macaques is thicker than that in mice. Because the diameter of the rat common carotid artery is similar to that of the monkey MCA, which may be difficult to induce thrombosis using low concentrations of FeCl_3_. Therefore, in our previous study, we initially screen the different concentrations and durations of FeCl_3_ application in rat common carotid artery [27]. The optimal conditions were obtained before we applied to the MCA in NHPs.

MRI has become a common way to detect intracranial infarcts in the acute and subacute phase of ischemic stroke [28, 29]. Previous studies have documented that the infarct regions after occlusion of the MCA-M1 segment mainly involve the temporal cortex, caudate nucleus, and putamen regions of the brain by MRI [30]. While occlusion of the MCA-M2 segment only causes a small infarct in the temporal cortex with mild neurological impairment involving the corresponding limbs and get faster recovery [31]. Currently, it remains challenging to produce an ideal ischemic stroke model in NHPs with a relatively large infarct size, significant neurologic impairments, and a low mortality rate. Accordingly, we used FeCl_3_ to induce the MCA-M1 thrombosis in NHPs. In this model, the reproducible infarct regions involving in the caudate, globus pallidus, putamen and cortex area were observed, which was the model induced by a microclamp in NHPs [32]. However, compared with the transorbital approach or transcranial operation for placement of a microclamp in MCA to induce stroke, our method did not require removal of the eyeball and avoided the MRI artifacts due to surgical clip placement. The infarct volume in our study was similar to that of the stroke induced by balloon to occlude the M1 segment of the MCA in NHPs [33]. Still, our survival rate was much higher than balloon method (50% mortality). The potential reason for the high mortality rate is because of the risk of vessel rupture when the balloon is inflated after entering the MCA. Our NHP stroke model could be evaluated by both neurobehavioral testing and MRI, which is in accordance with the updated Stroke Therapy Academic Industry Roundtable (STAIR) recommend-dations [34].

Neurological impairment persists for up to 2 weeks after stroke in mice [35, 36]. However, sensorimotor deficits in patients with severe cerebral infarction, hemiplegia, and other neurological dysfunctions often last up to 6 months. In the model described in this study, severe neurological impairment was observed 24 hours after MCAO, which could last at least for 3 months. The sensorimotor impairment includes weakness of the upper and lower limbs, and rotating problem. The behavioral outcomes in our study are in agreement with previous work by Ben who induced MCAO in macaques by electrocoagulation [37]. Compared with MCAO induced by electrocoagulation, our method also could precisely control the position of induced thrombus in the MCA without damaging the peri-arterial tissue, which more closely resembles the clinical setting. The FeCl_3_-induced infarct and sensorimotor deficits can be observed at the chronic stage, suggesting that this model is suitable for observing the long-term effects of medication and rehabilitation.

In the current study, we found that FeCl_3_-induced NHP thrombotic model did not cause the autolysis of the thrombus, and the thrombosis could be precisely positioned. Therefore, the successful rate in inducing ischemic stroke in NHPs is very high. While the autologous thrombus-mediated stroke model developed by Kito had a failure rate of 21% [14]. Although the injected autologous thrombus can lead to robust occlusion of the MCA and produce contralateral motor and sensory disturbances related to the infarct volume, the accuracy of the occlusion position is limited. Compared with the autologous thrombus injection model, our method had higher success rate, precisely position and very low mortality rate. Autologous thrombus injection model could be affected by the distribution of thrombi, the degree of spontaneous recanalization, and onset of recanalization, making the experimental data inconsistent and poorly controllable. Emboli entering non-target vessel segment was possible, such as M2 segment, which may be the reasons for the failure of autologous thrombus injection method. If the thrombus stays upstream of the M1 segment, it may cause a larger area of cerebral infarction, resulting in higher mortality rate.

One of the advantages is that FeCl_3_-induce stroke model can be used for thrombolysis studies. The main mechanism of FeCl_3_-induced vascular thrombosis is partial detachment of local vascular endothelial cells, and basement membrane components into the circulating system [38]. At the same time, a large number of Fe-filled spherical bodies appear on endothelial cells. Platelets and tissue factor can then attach to these spheres, forming aggregates that trigger thrombin reaction and thrombus formation. FeCl_3_-induced thrombosis is sensitive to anticoagulant and antiplatelet drugs [39]. Our previous study showed that FeCl_3_-induced thrombosis in the mouse MCA could be dissolved by human tissue-type plasminogen activator (rt-PA) in a short time after embolism[40]. Consistently, another study confirmed that FeCl_3_-induced thrombi in mouse carotid arteries could be dissolved by thrombolytic drugs [41]. We demonstrated that the thrombosis induced by 50% FeCl_3_ could be dissolved partly by rt-PA in rat common carotid artery [27]. These results suggest that this stroke model is reliable for both modeling and therapeutic studies.
